# Carrier Recombination in Nitride-Based Light-Emitting Devices: Multiphonon Processes, Excited Defects, and Disordered Heterointerfaces

**DOI:** 10.3390/nano14131072

**Published:** 2024-06-23

**Authors:** Grigorii Savchenko, Evgeniia Shabunina, Anton Chernyakov, Nadezhda Talnishnikh, Anton Ivanov, Alexandr Abramov, Alexander Zakgeim, Vladimir Kuchinskii, Grigorii Sokolovskii, Nikita Averkiev, Natalia Shmidt

**Affiliations:** 1 Ioffe Institute, 26 Politekhnicheskaya, St Petersburg 194021, Russia; jenni-85@mail.ru (E.S.); bs@mail.ioffe.ru (A.A.); vladimir.kuch@mail.ioffe.ru (V.K.); gs@mail.ioffe.ru (G.S.); averkiev.les@mail.ioffe.ru (N.A.); natalia.shmidt@mail.ioffe.ru (N.S.); 2 Submicron Heterostructures for Microelectronics Research and Engineering Center RAS, 26 Politekhnicheskaya, St Petersburg 194021, Russia; chernyakov.anton@yandex.ru (A.C.); nadya.fel@mail.ru (N.T.); a-e-ivano-v@yandex.ru (A.I.); zakgeim@mail.ioffe.ru (A.Z.); 3 Department of Electronics, Saint Petersburg Electrotechnical University «LETI», 5, Professora Popova St., St Petersburg 197376, Russia

**Keywords:** external quantum efficiency, efficiency droop, multiphonon recombination, nitrides, LED, random alloy fluctuations, band fluctuation potential

## Abstract

We study recombination processes in nitride LEDs emitting from 270 to 540 nm with EQE ranging from 4% to 70%. We found a significant correlation between the LEDs’ electro-optical properties and the degree of nanomaterial disorder (DND) in quantum wells (QWs) and heterointerfaces. DND depends on the nanoarrangement of domain structure, random alloy fluctuations, and the presence of local regions with disrupted alloy stoichiometry. The decrease in EQE values is attributed to increased DND and excited defect (ED) concentrations, which can exceed those of Shockley–Read–Hall defects. We identify two mechanisms of interaction between EDs and charge carriers that lead to a narrowing or broadening of electroluminescence spectra and increase or decrease EQE, respectively. Both mechanisms involve multiphonon carrier capture and ionization, impacting EQE reduction and efficiency droop. The losses caused by these mechanisms directly affect EQE dependencies on current density and the maximum EQE values for LEDs, regardless of the emission wavelength. Another manifestation of these mechanisms is the reversibility of LED degradation. Recombination processes vary depending on whether QWs are within or outside the space charge region of the p-n junction.

## 1. Introduction

The complexities of charge carrier recombination processes in nitride-based light-emitting devices (LEDs) are crucial in understanding various related physical phenomena. These phenomena include the efficiency droop effect, characterized by a decrease in efficiency with increasing injection currents, a reduction in efficiency due to higher indium or aluminium content (known as the “green gap” issue), notably low efficiency levels of UV LEDs using AlGaN alloys, reversible degradation processes, the short lifespan of laser diodes, and the creation of defects under injection current [1,2,3,4,5,6,7,8,9,10].

The most widely adopted approach for describing recombination processes in LEDs is based on the simple ABC model, where A represents the Shockley–Read–Hall (SRH) coefficient, B stands for the radiative coefficient, and C denotes the Auger coefficient [11]. The ABC model operates with the SRH recombination model. The SRH net recombination rate is intrinsically generic, because it represents only the occupation statistics of electrons and holes without accounting for any particular capture mechanism [6]. Furthermore, the ABC model assumes relatively ideal heterointerfaces and uniform distributions of electrons and holes in the multiple QWs. The observed deviations in real LED characteristics from those calculated within the framework of the ABC model are often attributed to imperfections in technological processes and such effects as the Stark effect, low hole concentration, high dislocation density, Auger recombination, and random alloy fluctuations (RAFs) [2,5,7]. However, during analysis, only a subset of these factors is typically considered, leaving others unexplored.

In some theoretical and experimental studies, it has been noted that the idealization of material properties leads to the neglect of fundamental properties of nitrides, such as dominant traps, which have electric-field-dependent capture cross-sections [6], as well as complex internal organization and a variety of forms of nanomaterial arrangement (NA), which are attributed to their domain (columnar) structure. Nitrides are low-dimensional, inhomogeneous nanomaterials. The various forms of NA are influenced by growth conditions, the properties of the buffer layer [12,13], and other factors such as domain size and their tilt, rotation, and coalescence. Both NAs and RAFs manifest themselves in features of the surface morphology [14,15]. According to [13,16], these features are characterized by the degree of nanomaterial disorder (DND). So, in further discussion regarding DND, we will understand it as the nanoarrangement of domain structure, RAFs, and the presence of local regions with disrupted stoichiometry of alloy composition. It is shown in [17] that an increase in the DND corresponds to a transition from 2D to a 3D growth mode accompanied by a deterioration in the electrical and optical properties of layers and LEDs. However, there is still a lack of a unified system of criteria for assessing the quality and DND of light-emitting structures to ensure high efficiency and lifespan.

The significant role of disorder effects in nitrides in understanding the physical processes determining the parameters of LEDs and the observed phenomena was highlighted in [1,17]. In particular, disorder is manifested in effects such as weak rectifying properties of p-n junctions, S-shaped temperature dependence of electroluminescence (EL), phonon replica in photoemission spectra, indirect phonon-assisted recombination mechanisms, and discrepancies between simulated and observed values of turn-on voltage in commercial LEDs. Furthermore, significant attention is given to RAFs [1,8,9,12], as they are more pronounced in nitrides. When analysing recombination processes and discussing the experimental results obtained in our study, we also take into account the aforementioned manifestations of disorder and RAFs in LEDs’ operation.

Furthermore, we have taken into account that DND in nitrides lead to the existence of local regions with disrupted stoichiometry, including an excess concentration of atoms or ions of metallic (In, Ga, Al) components [9,18]. Similarly to single SRH defects [19], they can also induce a disruption of the lattice arrangement symmetry. This leads to bond deformation and consequently results in the presence of excess elastic energy, leading to the formation of so-called excited defects (EDs) [20,21]. We assume that in nitrides, the local concentration of EDs may exceed the concentration of SRH defects by orders of magnitude, as RAFs in local areas of the layers can reach several percent [9].

It is well known that charge carrier capture processes, including those by EDs [19]; their vibrational relaxation in the lattice after capture; as well as multiphonon defect recombination in QWs [22] can complicate the charge carrier transport and recombination processes in nitrides.

Please note that the features of recombination processes involving EDs have been studied in gallium arsenide [20,23,24], while in nitrides they have been scarcely investigated, despite the fact that the Huang–Rhys factor, reflecting the excess elastic energy of deformed bonds in the lattice, is higher in nitrides than in arsenides [22].

Alongside the approaches developed in the ABC model, there are other approaches grounded in atomic [8] and localization landscape [9] models. These models incorporate considerations of quantum tunnelling in disordered nanomaterials, fluctuations in the thickness of multiple QWs in both vertical and lateral directions [6,9], indium penetration in GaN barriers [8,12,18], the disorder of heterointerfaces, and the band fluctuation potential (BFP) associated with random indium fluctuation [12].

Several studies have demonstrated that the intricate dynamics of carrier transport and recombination processes, as well as carrier localization, correlate with the crystal quality of blue LED structures [13,14,16,17]. Furthermore, an influence has been revealed of the surface morphology of layers, LEDs, and laser diode structures on their optical and electrical properties. It has been demonstrated that this correlation is not accidental [12,13,14,16,17,25]. It arises from the features of nitrides listed above. Additionally, in typical nitride-based LED structures, to prevent alloy decomposition, the QW width does not exceed 3 nm. Meanwhile, the root mean square roughness values in materials with high DND can approach 2 nm, making it difficult to obtain well-structured QWs.

In [9], estimates are provided of the BFP associated with random indium fluctuation [12], such as 40–50 meV for an alloy with 20% indium content. However, experimental data on the values of BFP for LEDs emitting in different spectral ranges are practically absent. This prevents the assessment of the contribution of RAFs to the recombination processes and parameters of LEDs with varying DND. Thus, the significant and still poorly understood role of DND in the operation of nitride-based LEDs to date does not allow for the creation of a unified theory of carrier transport and recombination. Similar challenges exist in the field of simulation of nitride-based light-emitting devices [1]. In this regard, a more thorough and deep analysis of experimental data appears to be the most appropriate approach. Existing models that account for the noted low-dimensional properties of nitrides are complex and do not allow for practical use as easily as the ABC model. It appears that in low-dimensional nitrides grown under non-equilibrium conditions and having different DND, it is challenging to establish unambiguous strong dependence between device parameters and growth conditions. A more accessible approach is to utilize correlations between device parameters and growth conditions. This approach may allow for the determination of quality criteria for LED structures.

In this work, we attempted, based on a previously established correlation between the electrical properties of LEDs and the DND, to experimentally determine to what extent the noted features of nitrides complicate the carrier recombination processes, affect the parameters of emitting devices, and participate in phenomena such as efficiency droop and green gap.

## 2. Materials and Methods

### 2.1. Experimental

In this study, we examined commercial blue, green, and UV LEDs. These LEDs, each with a 1 mm^2^ active area, underwent flip-chip packaging. The external quantum efficiency (EQE) of blue LEDs ranges from 50% to 70% at wavelengths between 450 and 460 nm, while green LEDs exhibit EQE ranging from 13% to 37% at wavelengths between 500 and 530 nm. The lowest EQE values (3–7%) are observed in UV LEDs at wavelengths between 270 and 280 nm.

Current–voltage (I-U) characteristics, EL spectra, and EQE of LEDs were measured across a temperature range of 50–400 K. I-U characteristics were obtained using a KEITHLEY 6487 power source (Cleveland, OH, USA). EQE measurements were conducted on LEDs at temperatures ranging from 100 to 450 K, employing an Optronic Laboratories OL770-LED system 141 (Orlando, FL, USA) with an OL ISA-670 integrating sphere [26]. Optical power was determined using a THORLABS DET02AFC/M photodetector (Newton, NJ, USA). EQE dependencies on current were measured in both direct-current mode at j < 30 A/cm^2^ and pulsed modes (100–300 ns pulses at 100 Hz) at j > 30 A/cm^2^, provided by an Agilent 8114A generator (Santa Clara, CA, USA). The optical signal was recorded using a high-speed photodetector THORLABS DET02AFC and a Tektronix TDS3044 oscilloscope (Beaverton, OR, USA). The emission spectrum of the LEDs was controlled using an OL 770-LED UV-VIS spectroradiometer. Calibration coefficients for each type of LED were obtained using a spectroradiometer with an integrating sphere OL 770 LED to determine absolute efficiency values at room temperature in accordance with the recommendation [27,28]. The temperature range was set using a Janis CCS-450 (Janis Research Co Inc, Woburn, MA, USA) cryostat with an optical window Janis CCS-450.

Atomic force microscopy (AFM) measurements were performed using an Ntegra AURA setup (NT-MDT, Moscow, Russia) with an HA_FM cantilever. The scanning speed was approximately 1.3 μm/s, and the cantilever had a stiffness coefficient of 3.5 N/m, a radius of curvature less than 10 nm, and a scanning field size of 256 × 256 points.

### 2.2. Employed Approaches

In addition to the concepts highlighted in the introduction, our analysis of the I-U characteristics of LEDs was based on the notion of a close relationship between the electrical properties of layers in nitride-based device structures and their NA. This connection is linked to the nature of NA and is supported by previously obtained experimental confirmation [16,17]. As some of these experimental works were carried out quite a while ago, alongside references, we provide several crucial experimental results.

During the first decade of this century, a significant amount of research was directed toward comprehending the intricate internal organization of nitrides. As a result, it was qualitatively demonstrated that the electrical and optical properties of GaN layers and the alloys based on them are intricately connected with the growth regime of the domain buffer layer and the morphology (topography) of the layer growing on it [12,13,14,17]. In this case, the surface topography was determined using AFM methods. It is worth noting that concepts regarding the self-organization of various materials with a complex internal structure, the reflection of their properties in surface morphology, and the quantitative assessment of these properties through multifractal analysis methods began to develop somewhat earlier [29,30]. The basis of multifractal analysis lies in the concept of a material grown in a self-organizing mode, when an exchange of matter, energy, and information with the environment occurs. As a result, nanomaterials containing spatial nano- or microstructures, such as domain structures, are formed. The properties of such materials are determined to a greater extent by the nature of the interaction between these structures, rather than the sum of the individual parts’ characteristics [29]. The choice of multifractal parameterization for the quantitative determination of the organizational features of semiconductor nanomaterials is grounded in the observation that the actual structures of materials are stochastic fractals, meaning they are self-similar only on average. The practical application of multifractal formalism became feasible with advancements in statistical and information physics, set theory—encompassing digital sets—and symmetry theory. For over 20 years, the methodology of multifractal parameterization for material structures has been successfully employed in the field of metals materials science [31]. There are many computer programs and multifractal parameters available to facilitate the quantitative characterization of the organizational features of spatial structures in various materials. We employed the informational interpretation of multifractal formalism using the computer program MFRDrom [30]. This program, when processing a digital set corresponding to the surface topography obtained by AFM methods, enables the generation of a spectrum of generalized entropies (Rényi information dimensions D_q_, where higher values indicate a higher level of entropy and poorer self-organization of the material). Furthermore, one can define the parameter ∆_p_, which reflects the degree of local symmetry disruption for the overall configuration of the structure under study. An increase in the absolute value of ∆_p_ indicates the violation of local symmetry and an increase in DND.

**Figure 1:**
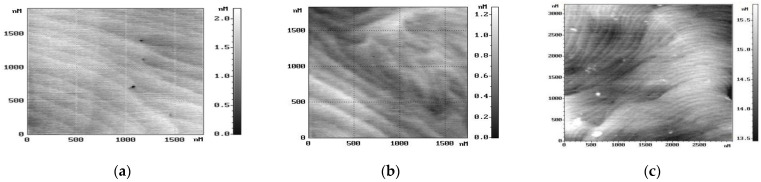
Surface topography of GaN layers obtained using AFM. ∆*_p_* and electron mobility values: (**a**) 0.320, 600 cm^2^V^−1^s^−1^; (**b**) 0.330, 400 cm^2^V^−1^s^−1^; (**c**) 0.360, 55 cm^2^V^−1^s^−1^.

**Figure 2:**
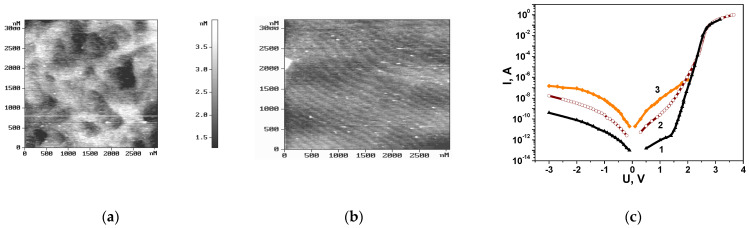
Surface topography of InGaN/GaN structures with different ∆*_p_*: (**a**) 0.355. The white line is the line along which the roughness profile was determined; (**b**) 0.330; (**c**) *I-U* characteristics of blue LEDs with different ∆_p_ at 300 K: curve 1—0.330; curve 2—0.348; curve 3—0.355.

The values of the parameter ∆_p_ were determined by processing data from a digital set corresponding to the surface topography of GaN layers and InGaN/GaN light-emitting structures using AFM. Data processing was performed using MFRDrom. A dedicated software program [30,31] enabled us to establish a correlation between the electrical properties of the layers and structures with ∆_p_ (Figure 1 and Figure 2). GaN layers and InGaN/GaN light-emitting structures were grown at the Ioffe Institute. In this case, multiple options for the growth modes of buffer layers were employed.

Figure 1 shows the surface topology of GaN layers obtained via AFM at the Ioffe Institute. The layers have similar dislocation densities and an electron concentration of $\left(0.5-1\right)\cdot 10^{17}cm^{-3}$. The surface topography of GaN layers illustrates a transition in growth mode from 2D to 3D, corresponding to a change in the growth mode of the buffer layer from (a) to (c), as shown in Figure 1. These changes are accompanied by an increase in the values of the multifractal parameter ∆_p_, indicating an escalation in the DND. Moreover, the electron mobility decreases by an order of magnitude, and the temperature dependence of mobility suggests an increase in the concentration of scattering centres and a transition to hopping conduction [12].

The I-U characteristics of blue LEDs, exhibiting the same changes in the growth regime of the buffer layer as observed in the GaN layers (Figure 1), are illustrated in Figure 2. In this case, the growth modes and design of the active region and p^+^ region were identical. The LEDs produced at the Ioffe Institute were synthesized via metal–organic chemical vapor deposition (MOCVD) on (0001) sapphire substrates using a conventional low-temperature GaN nucleation layer technique [18]. These structures consist of a 2.5 μm GaN buffer layer, 5 pairs of MQW InGaN/GaN (2/8 nm), a 15 nm p-AlGaN layer, and a 120 nm p^+^-GaN layer.

The surface topography of the InGaN/GaN structures (Figure 2) also indicates a shift in the regime of structure growth and an increase in the parameter ∆_p_. This correlates with a degradation in the quality of the p-n junction, an elevation in the non-ideality factor of the direct branch of I-U characteristics, an increase in tunnelling currents, and a rise in the concentration of charged centres in the SCR region of the p-n junction. According to the studies conducted in [32], the features of the reverse branches, observed in all nitride-based LED structures, are attributed to hopping transport of charge carriers. The packaging of these structures did not lead to a noticeable change in the observed correlation of the I-U characteristics and the current levels in the forward and reverse directions with the morphology of the structures under study. Thus, the obtained results allow for the utilization of the identified correlation to evaluate the quality and DND in the packaged LEDs. The identified correlation between the current–voltage characteristic and the DND of the nanomaterial appears quite natural, as disorder primarily results in random local changes in heterointerfaces. Simultaneously, the root mean square roughness of the alloy layers in the case of ∆_p_ = 0.355 exceeds 2 nm, as per the AFM data. As the thickness of the QWs in nitrides is less than 3 nm, with such roughness, it is impossible to ensure efficient radiative recombination. Furthermore, these heterointerface fluctuations may result in the local accumulation of gallium and indium in such regions, owing to their large tetrahedral radii.

## 3. Results

### 3.1. I-U Characteristics; Deviation from Standard Shockley–Read–Hall Model—Correlation between Efficiency and Heterointerface Disorder

**Figure 3:**
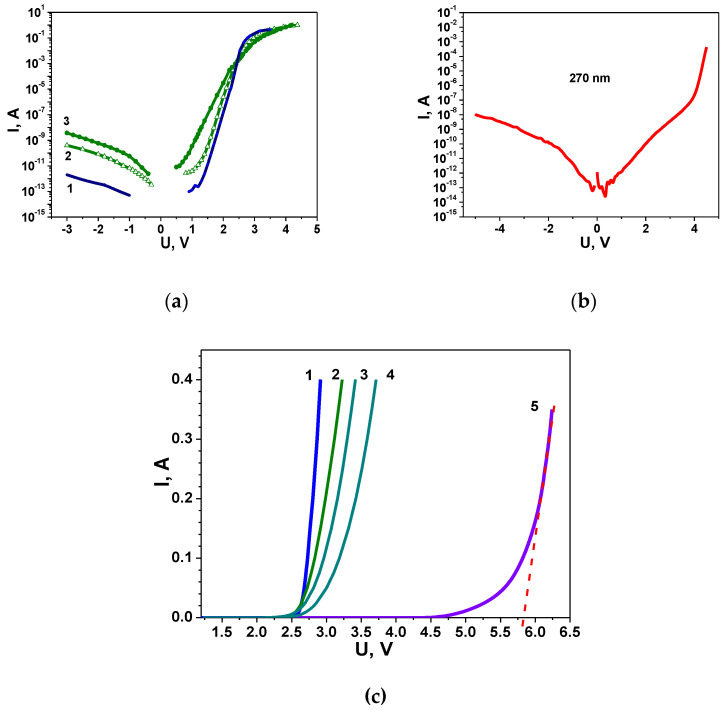
I-U characteristics of LEDs at 300 K: (**a**)—blue with EQE of 70% (curve 1) and green with EQE of 30% (curve 2) and 12% (curve 3); (**b**)—UV; (**c**)—blue (curve 1), and green with EQE of 30% (curve 2), 16% (curve 3), and 12% (curve 4); UV (curve 5)—dashed line demonstrates graphical determination of the threshold voltage.

Let us examine the experimental current–voltage characteristics of commercial LEDs emitting at different wavelengths and exhibiting significantly different EQE values. The I-U characteristics of LEDs are depicted in Figure 3 on both a semi-logarithmic and linear scale.

It is noteworthy that the shape of the I-U characteristics on a semi-logarithmic scale is shown to be typical for nitrides [32,33,34]. Note that both the forward and reverse branches of the current–voltage characteristics deviate from the characteristics of an ideal p-n junction obtained within the framework of the standard Shockley theory. In the direct branches of all current–voltage characteristics (Figure 3a,b), regions with excess recombination currents of a tunnelling nature are observed, characterized by a non-ideality factor n > 2. It is evident that the voltage ranges in which tunnel currents are observed, as well as their values, significantly decrease with the increase in LED efficiency. This pattern is described in [35] and provides a good reflection of DND. As mentioned in Section 2, we previously observed a correlation between tunnel current levels, NA, and DND in blue LEDs grown on buffer layers formed in different growth modes [17]. The generality in the observed trends suggests that the efficiency value is significantly influenced by the DND of LED structures. In the study in [36] on silicon p-n junctions, it was demonstrated that n > 2 can be indicative of an abrupt p-n junction. This provides grounds to believe that, in our case as well, in nitride-based LEDs, in addition to the tunnelling nature of the current, we can speak of an abrupt p-n junction. This assumption is further supported by comparing the values of the currents in the forward and reverse branches of the I-U characteristic at small biases: the forward and reverse currents are only slightly different in magnitude, indicating a p-n junction with poor rectifying properties. It is worth noting that in UV and green LEDs, with minimal efficiency, the rectifying properties are the worst (Figure 3).

The dependence of the reverse current on voltage is qualitatively characterized by an exponential function, while according to the Shockley–Read–Hall (SRH) theory, the current $I\propto \sqrt{U}$. A different type of dependence indicates that the current in nitride-based LED structures has a nature distinct from the SRH recombination current. High reverse current values, in addition to the poor quality of the p-n junction itself, also indicate the presence of cations in the SCR. According to the research presented in [32], such a form of the I-U characteristic is attributed to carrier hopping transport associated with non-uniformly distributed cations in the SCR of the p-n junction. Note that cations can be present at heterointerfaces even under forward bias (at small biases). They can change their charge state as the current increases in the forward direction due to interaction with tunnelling charge carriers.

A comparison of the current–voltage characteristics of UV, blue, and green LEDs (Figure 3) allows us to conclude that one of the reasons for the low EQE values of UV LEDs is the high concentration of charged centres in the active region and the high level of DND. Additionally, previous experiments [18] have shown that the blurring of heterointerfaces is a result of the diffusion penetration of In, Ga, or Al into the barriers. All of these factors are manifested in the shape of the I-U characteristic. Hence, the analysis of the full I-U characteristics of LEDs provides the opportunity to extract a wealth of valuable information beyond the typically used data on the tunnelling component of the current. In particular, this allows assumptions to be made about the quality of heterointerfaces and multiple QWs. Thus, LEDs with poorly structured QWs and barriers, where efficient radiative recombination cannot be realized, can be identified by electric measurements.

Important information is carried by the forward branch of the current–voltage characteristic on a linear scale (Figure 3c). The threshold voltage u_th_ corresponding to an open p-n junction and flat zones is determined by extrapolating the linear section of the current–voltage characteristic to the intersection with the voltage axis [37]. For green LEDs, it ranges from 2.7 V to 3.3 V, for ultraviolet (wavelength 280 nm) it reaches 5.6 V. Noteworthy is the difference between the values of u_th_ and the sub-threshold voltage u_0_ corresponding to the maximum of EQE. For green LEDs (Figure 3a), this difference ranges from 0.46 V (LEDs with a maximum EQE of 30%) to 1 V (maximum EQE of 12%). For a UV LED (curve 5 in Figure 3c), it is maximum and amounts to 1.4 V, while for a blue LED (curve 1 in Figure 3c) this difference is close to zero. According to [33,37], this difference may be related to fluctuations in the band potential caused by RAFs.

Thus, the difference u_th_-u_0_ carries information about RAFs and the quality of the p-n junction [35]. The correlation described in Section 2.2 between the morphology of the structure and the I-U characteristic gives reason to believe that the difference u_th_-u_0_ is associated with the technological perfection of LEDs: it is small for more advanced LEDs and takes on large values in the case of LEDs with low efficiency.

Thus, there are two causes of high DND of LED structures: the first is associated with the conditions of 2D and 3D or step-flow growth mode, and the second is due to the conditions of indium or aluminium incorporation into the growing layer. Apparently, these processes are interconnected. Our assumptions are in line with the results of optical measurements presented in [15,38].

Thus, the analysis of the I-U characteristics of nitride-based LEDs with different emission wavelengths revealed more complex charge carrier recombination processes not reduced to simple interactions of single uniformly distributed SRH defects with charge carriers.

### 3.2. Multiphonon Processes—Manifestation in Electroluminescence Spectra; Excited Defects

**Figure 4:**
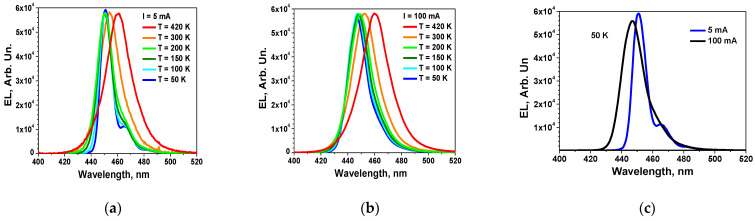
Electroluminescence spectra of a blue LED: (**a**)—5 mA at different temperatures; (**b**)—100 mA at different temperatures; (**c**)—5 and 100 mA at 50 K.

Another important fundamental property of nitrides, the high probability of multiphonon recombination, is manifested in the features of the temperature dependence of the I-U characteristics. It correlates with the one described in the framework of the semi-classical trap-assisted multiphonon tunnelling (TAT) model [22]. To understand the significant impact of the DND on the efficiency of LED structures, initially perceived as a formal correlation, we conducted measurements of the EL spectra of various nitride-based LEDs, as well as their EQE values as a function of pump current density. It turned out that multiphonon processes are also evident in the EL spectra. Electroluminescence spectra and their temperature dependences are presented in Figure 4.

The phonon replica at a temperature of 50 K is noteworthy (Figure 4a). The difference between the positions of the two maxima corresponds to the energy of the LO phonon (90 meV). An increase in temperature leads to a broadening of the spectrum, as does an increase in current. Please note that increasing the injection current from 5 mA to 100 mA at 50 K results in the same spectral changes as raising the temperature to 200 K while maintaining a constant current of 5 mA (Figure 4a,b). In this scenario, the spectral width at half maximum (FWHM) nearly doubles.

The variation in the EL spectrum with temperature at a current of 5 mA demonstrates well-known S-shape temperature dependencies, attributed, according to [1], to the significant role of DND in the recombination processes in InGaN/GaN QWs. From Figure 4b, it can be seen that at a current of 100 mA, there is a weak change in the electroluminescence spectra with temperature in the temperature range of 50–200 K, which is less pronounced compared to the expected variation from the Varshni formula. This allows us to assume that, during the interaction of injected carriers with excited defects, a phenomenon known as recombination-induced local defect heating occurred. The concept of EDs is developed in several studies and is extensively described in the monography in [21] and the review in [20].

According to [39], the presence of an electric field significantly influences the mechanism of multiphonon recombination. In this regard, it is important to verify whether the recombination processes in the QWs within the SCR (at u< u_th_) or outside it will differ. It is known from C-V profiling research [40] that at zero bias, typically only 2–3 QWs from the entire profiling are within the SCR. This circumstance turns out to be extremely important in the analysis of recombination processes.

### 3.3. Analysis of LED Efficiency—Distribution of EQE over Wavelengths; Band Fluctuation Potential

It is important to determine the contribution of the detected features of the NA of the active region of LED structures to phenomena such as the decrease in EQE with increasing injection current and the overall reduction in EQE with increasing concentration of In or Al.

**Figure 5:**
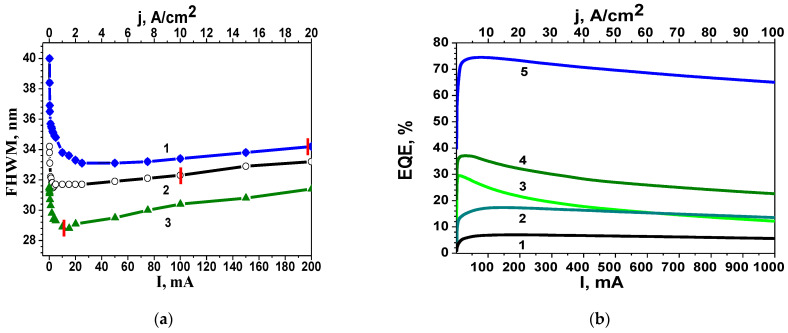
(**a**)—FWHM versus current at 300 K for green LEDs with different maximum EQE (1—12%, 2—16%, 3—30%) and different DND of heterointerfaces (DND worsens from 3 to 1). Red dashes mark the current corresponding to the u_th_; (**b**)—EQE of different LEDs (1—UV, 2—green with EQE of 12%, 3—green with EQE of 30%, 4—green with EQE of 38%, 5—blue) versus current density at 300 K.

For a more detailed analysis, it makes sense to study the dependence of the width of the EL spectra at half maximum (FWHM) on the injection current for green LEDs with different DND. This is shown in Figure 5a. Please note that the maximum of EQE corresponds to low voltages, i.e., the case of a non-open p-n junction. From Figure 5a, it can be seen that the EL spectrum narrows with increasing current to values of approximately 20 mA, which corresponds to a case when the QWs lie inside the SCR. It is in this area that an increase in EQE is observed (Figure 5b). At currents greater than 20 mA, the p-n junction is open, the QWs lie outside the SCR, the EL spectrum broadens (Figure 5a), and the EQE decreases (Figure 5b). Maximum efficiency for all LEDs is achieved at current densities less than 20 A/cm^2^. This corresponds to the case when QWs are located in the SCR of the p-n junction. From the above, it follows that a decrease in the efficiency of LEDs with increasing injection current is observed in a situation where the QWs lie outside the SCR. It can be assumed that such behaviour of the FWHM is associated with the presence of excited defects.

**Figure 6:**
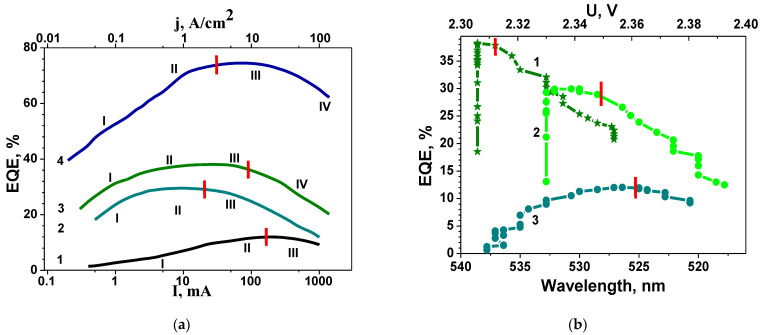
EQE versus current density at 300 K. Red dashes mark the current corresponding to the u_th_. (**a**) 1—green 12%; 2—green 30%; 3—green 38%; 4—blue 70%. (**b**) Distribution of EQE of green LEDs over wavelength at 300 K with efficiency of 38% (curve 1), 30% (curve 2) and 12% (curve 3). The injection current magnitude increases from left to right, ranging from 0.03 to 200 A/cm^2^.

The EQE dependencies of blue and green LEDs on injection current density is presented in Figure 6a. From the comparison of Figure 5a and Figure 6a, it follows that more advanced LEDs with greater efficiency correspond to a narrower EL spectrum. Along with the narrowing of the EL spectrum, as the current increases, the spectrum shifts to the short-wave region (see Figure 4). A comparison of the dependence of wavelength corresponding to the maximum of EL spectrum on current with the dependence of the EQE on current (Figure 6a) allows us to obtain the distribution of LED efficiency over wavelengths, as shown in Figure 6b. The red dashes mark the wavelengths at voltage u_th._

Efficiency increases with current for all LEDs before reaching the threshold voltage u_th_. When u > u_th_ (open p-n junction), efficiency starts to decline in all cases. At the same time, for efficient LEDs, four characteristic regions are distinguished in the current dependence (Figure 6a). At current densities greater than 5 A/cm^2^, measurements were carried out in pulsed mode.

Considering the variety of physical mechanisms leading to the charge carrier recombination in nitride-based LEDs, it should be expected that the recombination processes also depend on the electric field. This gives reasons to separately consider the processes that take place in the case when QWs are located inside the SCR of the p-n junction (at small biases, regions I and II in Figure 6a) and outside its borders (regions III and IV) [35]. The threshold voltage u_th_, marked on each curve in Figure 6 with red dashes, serves as a conditional boundary between these two states. Moreover, since we assume that the efficiency of LEDs is related to the perfection of the active region and the DND, it makes sense to separately consider relatively efficient LEDs (blue and green with maximum EQE of 30% and 37%: curves 2, 3 and 4 in Figure 6a and curves 1 and 2 in Figure 6b) and LEDs with low efficiency (green with EQE of 12%—curve 1 in Figure 6a, curve 3 in Figure 6b; UV with EQE 4–7%).

Let us first consider the efficient LEDs (curves 2, 3, and 4 on Figure 6a and curves 1 and 2 on Figure 6b). In region I, a rapid increase in EQE is observed with an increase in the injection current density. In the EQE distribution across wavelengths (Figure 6b), this region corresponds to vertical lines, meaning that the current increase does not lead to a shift in the spectra in the wavelength. In other words, optical transitions of a single type are observed in this region. This corresponds to the well-known tunnelling radiative recombination in QWs [41]. Note that since on this region u < u_th_, the QWs lie within the SCR.

With a further increase in current, the rise in EQE occurs more smoothly (segment II in Figure 6a). This indicates the emergence of a new source of losses. On the wavelength distribution (Figure 6b), this is manifested as a horizontal segment up to u_th_. That is, in this range, the emission wavelength changes with increasing current. At the same time, there is no distinctly pronounced type of optical transitions, i.e., well-organized QWs are absent. The extent of this region in Figure 6b makes it is possible to estimate the average BFP in the case where QWs lie within the SCR. For this, it is enough to convert the wavelength into energy. The narrower the plateau region is, the more advanced the LED is and the higher its efficiency. The corresponding estimates of BFP, as well as EQE for various LEDs, are presented in Table 1.

So, the change in the emission wavelength with the variation in injection current density can be linked to RAFs and imperfections at the heterointerfaces, leading to the emergence of new optical transitions and a corresponding shift in the emission wavelength. The extent of horizontal segments in the EQE distribution across wavelengths, expressed in the energy scale, allows the average magnitude of the BFP to be estimated. It is noteworthy that there is a correlation between the maximum efficiency of LEDs, the corresponding values of u_th_-u_0_, and the magnitude of the BFP within the SCR.

Therefore, the increase in all parameters of the LEDs listed in the table is accompanied by a decrease in maximum efficiency.

As the voltage increases above u_th_, the EQE begins to decline. However, initially (region III), this decrease is not as sharp. Region III in Figure 6a corresponds to an open p-n junction (u > u_th_) and a relatively smooth decrease in EQE with increasing current (see Figure 6a). In this range, the wavelength of the emitted light still changes with increasing current density (Figure 6b), but the efficiency begins to decrease. This indicates that the associated losses, usually attributed to RAFs and imperfections in the heterointerfaces, are intensified. Tunnel transport of injected carriers occurs in the fluctuation potential field. Since, in this situation, the QWs are outside the SCR, they are beyond the built-in electric field of the p-n junction, which provides directional carrier motion in the growth direction of the active region. That is the reason why charge carriers can move in the lateral direction (in the plane of the layers). In such a situation, the probability of charge carrier transport to extended defects, predominantly nonradiative recombination centres, increases. This accounts for the decline in efficiency.

A further increase in pump current (region IV in Figure 6a) leads to the fact that deep states of the fluctuation potential gradually become occupied, leading to a shift in transport mechanisms from lateral tunnelling to diffusion, which is typical for conventional injection in a forward-biased p-n junction. In this scenario, extended defects, such as dislocations and grain boundaries, begin to play a more prominent role. The interaction of these defects with injected carriers transforms them into active centres of predominantly nonradiative recombination, contributing to a more rapid decline in peak efficiency at high injection currents. In other words, at high current densities (non-equilibrium charge carrier density n > 10^18^), the activation of existing impurity complexes and defect generation under recombination-induced defect heating take place [20]. It should be noted that with a further increase in current, it is possible to activate an additional mechanism of efficiency droop associated with the crowding effect [42].

Next, let us turn our attention to LEDs with low efficiency (curve 1 in Figure 6a and curve 3 in Figure 6b). Here, the efficiency increases only slightly with increasing forward bias up to u_th_. Unlike efficient LEDs, this increase in efficiency is accompanied by a change in the emission wavelength from the beginning, i.e., from small biases (see Figure 6b). After opening the p-n junction, i.e., above u_th_, increasing the injection current leads to a decrease in efficiency, though not as sharp as in the case of efficient LEDs.

Please note that a similar type of distribution (curve 3 in Figure 6b) was observed earlier in samples grown under indium-enrichment conditions [43]. Such growth conditions lead to the formation of local regions enriched in In. The presence of such regions with excess Indium ions in the SCR is a source of nonradiative carrier losses and is typical for LED structures with low efficiency. Please note that the initial efficiency values of a green LED with regions highly enriched in indium are 100 times lower than those of a green LED with a slight Indium enrichment in QWs and at heterointerfaces (14% and 0.17%). This conclusion can be confirmed when comparing the difference u_th_-u_0_ for different LEDs (see Table 1).

Note that the change in wavelength (Figure 6b), accompanied by a decrease in efficiency at u > u_th_, is more significant than in the previous case. Table 1 provides estimates of the BFP at u > u_th_. Comparing the values of the BFP inside and outside the SCR allows us to assess the optimal growth conditions for matching the lattice constants of the buffer layer and the active region in the structure. If the BFP outside the SCR exceeds the BFP inside the SCR, it indicates that lattice mismatch-induced stresses are relieved before the active region begins to grow. Otherwise (green 4 in Table 1), the chosen growth modes did not result in a proper matching of lattice parameters prior to the initiation of active region growth. Please note that the lattice parameter matching and the penetration of indium or aluminium into the growing layers of the active region are closely related processes. The comparison between green 1 and green 2–3 (Figure 6b) allows us to conclude that the green gap issue is more determined by the conditions of indium introduction rather than just its concentration.

As noted, despite the low quality of the active region, an increase in current leads to some growth in efficiency with a simultaneous narrowing of the spectrum (see Figure 5a), indicating improved ordering of the heterointerfaces and greater alloy homogeneity. This effect can be explained by the presence of EDs, which can alter their coordination position in the lattice when interacting with injected carriers and phonons, thereby improving the ordering of the entire structure. It is important to note that this process is reversible: when the pumping current decreases, the FWHM returns to its original values. This process also occurs in efficient LEDs, but it is less pronounced since in these cases, the structures are initially more ordered (the BFP value is initially smaller). Reversible processes are possible due to the fundamental property of nitride materials—the excess elastic energy of alloy components in the lattice. Additional energy obtained during carrier recombination on EDs makes it possible to change the spatial position of alloy components in the lattice. Note that the EDs’ effect, manifested in the narrowing of EL spectra, occurs only at low current densities. Increasing the current above 20 mA causes the spectra to broaden for all LEDs under investigation (Figure 5a). It can be assumed that immediately after 20 mA, the spectrum broadening is associated with the filling of the BFP states. With a further increase in current (at non-equilibrium carrier concentrations greater than 10^18^ cm^−3^), the broadening occurs due to recombination-induced defect heating [20,21]. This constitutes two of the sources of carrier loss, contributing to a slower increase in peak efficiency.

**Figure 7:**
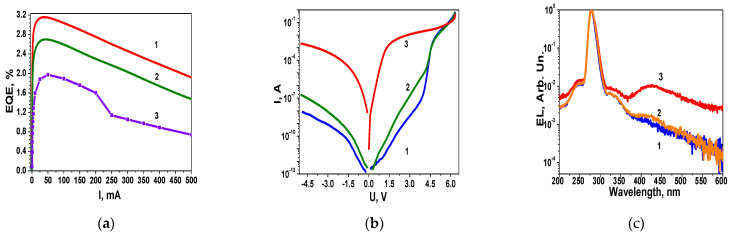
(**a**) EQE of UV LEDs versus current: 1—initial; 2—after recovery; 3—after 25 h of aging. (**b**) Evolution of I-V characteristics at aging: 1—initial; 2—after recovery; 3—after 25 h of aging. (**c**) EL spectra of UV LEDs at 1 mA: 1—initial; 2—after recovery; 3—after 25 h of aging. All dependencies presented were measured at 300 K.

Thus, we can identify two mechanisms of involvement of EDs in shaping the EL spectrum of LEDs: the first occurs at low current values and leads to spectrum narrowing and EQE growth; the second is observed at I > 20 mA and is associated with EL spectrum broadening and efficiency decrease. Both mechanisms are manifested in the change in the EL spectra of UV LEDs (Figure 7a) with increasing current.

### 3.4. Manifestation of Excited Defects in Aging and Degradation Experiments of UV LEDs

Additionally, the involvement of EDs is evident in aging experiments (Figure 7). At the same time, similar to blue and green LEDs [4], the reduction in EQE (Figure 7a) during aging is accompanied by a transformation of the I-U characteristics (Figure 7b curve 3). However, similar aging trends to those observed in blue and green LEDs are observed in UV LEDs at significantly shorter aging times, only 25 h at a current of 150 mA without a heat sink, whereas, for most blue LEDs, this typically occurs after several thousand hours. Simultaneously, the concentration of EDs increases by several orders of magnitude (as evidenced by the reverse-bias currents), and the rectifying properties of the p-n junction deteriorate, similar to what occurs in blue and green LEDs. Let us analyse in more detail the experimental results presented in Figure 7. The observed trends are accompanied by a change in the shape of the EL spectrum of the UV LEDs in Figure 7c, from curve 1 (initial) to curve 3 (after aging), measured at the same current of 1 mA.

The typical initial EL spectrum of UV LEDs in Figure 7c (curve 1) at a current of 1 mA demonstrates the phase separation of the alloy in the region 240–350 nm, confirming the conclusion drawn from the analysis of the forward branch of the I-U characteristics in the linear scale (Figure 3c) about the significant RAFs. Additionally, there is a broad defect band in the spectral range of 350–570 nm, typical for UV LEDs.

From Figure 7c, it can be observed that the shape of the spectrum in the narrow range of 270–280 nm remains unchanged during aging, while in the surrounding ranges of 200–250 nm and 320–350 nm, radiative recombination intensifies under current stress, accompanied by a broadening of the spectrum. These peculiarities of the EL spectra suggest that in the range of 270–280 nm, unlike the range of 200–250 nm и 320–350 nm, greater equilibrium in the alloy composition is observed.

The enhancement of radiative recombination after aging is also observed in the defect band region in the range of 350–570 nm. Therefore, the interactions between EDs and non-equilibrium charge carriers, which manifest during aging in defective regions, as indicated by the EL spectra, are sources of charge carrier losses, leading to a decrease in the efficiency of the EL peak. The observed phenomena are similar to those noted above for green LEDs with high DND. Another source of efficiency decrease in LEDs during aging is the disruption of the p-n junction (disrupted heterointerfaces), identified by the deterioration of the rectifying properties of the p-n junction and the increase in the leakage current level (Figure 7b, curve 3). The disrupted heterointerfaces worsen the perfection of multiple QWs and, consequently, the localization of charge carriers in multiple QWs. The disruption of heterointerfaces takes place at n > 10^18^ cm^3^. This occurs during aging under recombination-induced defect heating and the generation of EDs under the injection current [19]. Additionally, regions with ordered alloys may occupy a smaller area than disordered regions, which, coupled with the losses, results in low peak EQE values.

Unique features of the interaction between EDs and charge carriers are observed in degraded UV LEDs. The experiments demonstrate that, for some LEDs, the application of current pulses or exposure for 1 h at 80 mA with a heat sink leads to nearly complete restoration of both the I-U characteristics and the EL spectrum, along with the initial EQE values (curves 2 in Figure 7a–c). However, the lifespan of the restored LEDs increases insignificantly, and it varies for LEDs from the same batch. Thus, the presence of local regions with disrupted stoichiometry is a significant cause of the reduced lifespan of LEDs as well as one of the reasons for the ambiguous prediction of the maximum lifetime of nitride-based LEDs using aging test data.

## 4. Discussion

It is worth noting that the experimental data presented by us on the I-U characteristics in Figure 3 and the dependencies of EQE on the current density of LEDs in Figure 6, emitting in different spectral regions and significantly differing in EQE values, are quite typical and closely resemble those published in numerous articles by various researchers. The main distinction lies in the analysis of these results. We conduct a comparison of the electrical and optical properties of LEDs, identifying common patterns and differences.

For instance, in the analysis of I-U curves, we do not limit ourselves to the examination of only the forward branch and tunnelling current component, as commonly demonstrated in most works [7,34]. As a result, we manage to uncover important information encoded in the features of the reverse branch of I-U curves, indicating the presence of non-uniformly distributed cations in the p-n junction, the QWs, and at heterointerfaces. This leads to a hopping carrier transport mechanism [32]. Importantly, this is a common feature across all nitride-based LEDs, evident not only from our experimental results but also from those presented in [32].

However, noticeable differences exist. Low EQE values of LEDs correlate with an increase in reverse bias current, i.e., with a rise in cation concentration, as well as with an increase in the tunnelling current component under forward bias and a deterioration in the rectifying properties of the p-n junction.

The observed correlation suggests that such centres may act as a source of charge carrier losses during the operation of LEDs under forward bias. Are these centres defects within the Shockley–Reed–Hall (SRH) model? The temperature dependencies of the forward branches, as presented in [22] and in our work [44], do not align with SRH behaviour. Furthermore, in [39], it is demonstrated that the peculiarities in the temperature dependencies of the forward branches of I-U curves in LEDs reflect the involvement of multiphonon recombination processes induced by the presence of excited defects. In this context, atoms and metal ions in A^3^B^5^ compound semiconductors, due to their large tetrahedral radii, may contribute to lattice symmetry disruption, but they are not the sole cause.

Moreover, the investigation of frequency dependencies of the spectral density of low-frequency noise (S_I_) in blue, green, and UV LEDs [44] has shown that the form of S_I_~1/f dependencies over a wide frequency range reflects processes in a system of closely spaced defects. The segment of dependencies at frequencies above 100 Hz, which lacks frequency dependence and reflects the prevalence of recombination processes involving SRH centres, is absent. The previously established correlation (see Section 2) between the shape of I-U curves and the level of DND at heterointerfaces and QWs also suggests the involvement of EDs in recombination processes.

It is well established from experimental data [8,9] that random fluctuations in the composition of nitride alloys in local areas within QWs can vary by several percent or more. As a result, in such regions, there may be a deviation from the stoichiometry of the alloy, and the local concentration of components can exceed 10^19^ cm^−3^, significantly surpassing possible concentrations of SRH centres. Moreover, these components disrupt the lattice symmetry and are in an excited state. The transition in the growth mode of LED structures from 2D growth to 3D growth also leads to the emergence of local areas with EDs. This is evident from atomic force microscopy (AFM) data on the increase in the root mean square surface roughness of layers when the growth mode changes from 0.2 nm to 2 nm in a 10 × 10 μm^2^ scan.

It is well known [20,21,45] that an excited defect represents a system of “deep centre + local vibrations”. The key feature of EDs is that for some time after capturing a charge carrier by such a defect, strongly excited local vibrations exist near the deep centre. This circumstance determines several distinctive properties of EDs:

1. For some time after capture, an excited defect possesses excess energy concentrated in the form of local vibration energy.
2. After this period, energy relaxation occurs, causing the deep centre to transition to a quasi-equilibrium state, with its position in the lattice potentially differing from the initial position. In other words, such defects have the ability to change their coordination position in the lattice.
3. With sufficient excitation of the crystal (optical or injection), the activation energy responsible for defect diffusion can be significantly reduced, leading to the ability of EDs to migrate over significant distances in the crystal.

The features of nitrides and the alloys based on them, including the presence of capture centres with excess elastic energy associated with a large difference in the tetrahedral radii of components, as well as relatively large local composition fluctuations, provide these materials with higher values of the Huang–Rhys factor compared to, for example, arsenides, which determines the relationship between the electronic and vibrational subsystems [22]. All of this allows us to hypothesize that it is precisely in nitrides where the concentration of EDs will be sufficiently high.

The electronic relaxation mechanism will prevail over the lattice one in situations where the semiconductor has a high density of free electrons, especially under optical or electrical excitation. In this case, the presence of EDs leads to a faster transition of the system to an equilibrium state.

In [20], it was noted that EDs have the ability to change their coordination position in the crystal lattice more significantly than regular defect states, for which only jumps between neighbouring potential minima are allowed (recombination-enhanced diffusion).

Thus, the formation and subsequent relaxation of EDs, as well as the processes associated with them, can significantly influence the properties of the entire physical system, especially under excitation.

The change in roughness at heterointerfaces, resulting from the presence of excess metallic components in the alloy [8,9], with an RMS close to 2 nm comparable to the QW width, makes it challenging to achieve efficient radiative recombination of charge carriers in the QW. Clearly, to attain high EQE values with a narrow QW width, it is crucial to maintain low RMS values across the entire emitting structure area. However, achieving such control is labour-intensive for practical applications.

Nevertheless, monitoring the complete I-U characteristics, which carry integral information about the density of EDs along with the identified correlations, allows for a rough estimation of the contribution of DND to the electrical and optical properties of LEDs.

The experimental results of the investigation into the optical properties of LEDs, as presented by us, suggest the involvement of EDs. Importantly, the shape of the EQE dependencies on current density for all investigated LEDs in both semi-logarithmic and linear scales does not differ significantly from those published in numerous publications. The fundamental difference lies in the fact that we do not predefine known recombination mechanisms but, by highlighting characteristic regions of EQE changes with current density, attempt to elucidate which mechanisms could lead to the observed features in the experimental dependencies. Additionally, we considered that, according to C-V profiling [40], only a portion of the QWs are within the SCR of the p-n junction. By marking the values of current density at which the p-n junction opens on the dependencies of EQE on current density with red lines (Figure 6a), we found that the changes in the character of EQE dependencies on current density are associated with the location of the QWs in the active region of the p-n junction or outside it. This result is quite expected, as according to [7,39], the presence of an electric field significantly influences the mechanism of multiphonon carrier capture by EDs.

To clarify the mechanisms responsible for the observed features of the experimental dependencies, we further analysed the changes in the behaviour of the FWHM spectra of green LEDs’ luminescence with various DND values (Figure 5a) as the current increased. It has been found that in QWs located in the SCR, in the range of j < 2 A/cm^2^, a narrowing of the FWHM is observed with the increase in current density, while in the range of j >2 A/cm^2^, broadening of the FWHM occurs. These data indicate that the interaction of charge carriers predominantly occurs with fluctuations in the alloy composition and confirm the theoretical insights and experimental data of the studies in [8,9] regarding the significant contribution of RAFs to the electrical and optical properties of nitride-based emitting devices.

It should be noted that we arrived at this conclusion using simpler and more accessible experimental methods than those used in the referenced works. Furthermore, the methods used allowed us to identify two mechanisms of carrier loss caused by their interaction with EDs, rather than with SRH defects, as assumed in numerous studies [2,5,6,7]. Carrier losses caused by the involvement of these mechanisms result in a very slight increase in EQE of 1–2% with a several-fold increase in j in the range of j > 2 A/cm^2^.

Important information about the presence of local regions with disrupted stoichiometry is revealed by changes in the FWHM spectra of green LEDs with various DND values (Figure 5a) and by changes in the EL spectrum with increasing the current of UV LEDs from 1 mA to 20 mA (Figure 7c) with high values of DND and BFP in the MQWs located in the SCR. Furthermore, it is shown that a low DND with a high indium content allows for the production of green LEDs with an EQE of 37%, whereas LEDs with higher DND and BFP but lower indium content demonstrate an EQE of 10–12% (Figure 6b). Thus, the reason for the decrease in EQE lies not so much in the high indium content as in the DND, including the optimal mode of indium introduction.

Unlike numerous studies [2,5] explaining EQE droop by the increase in the contribution of Auger recombination, our experimental results have shown that the dependence of EQE on current density has at least two regions: the first one starting from the opening of the p-n junction, and the second one at j > 30 A/cm^2^. The decrease in EQE at the first region is caused by carrier losses during tunnelling in the lateral fluctuation potential field. This mechanism has been discussed in several studies [1]. It should be noted that in these studies, the value of BFP was not estimated. The analysis of the EQE distribution across wavelengths allowed us to estimate the magnitude of BFP in the QWs located inside and outside the SCR (data presented in Table 1 for all investigated LEDs). This is accompanied by a slight increase in the FWHM values of LED luminescence spectra.

As the states of the BFP fill up (with further increase in current), a change in the emission wavelength allows us to suggest the generation of EDs in local areas as a result of local heating under the recombination-induced defect heating mechanism, which suppresses radiative recombination in such localized areas. Also, the generation of defects at n > 10^18^ cm^−3^ takes place [20].

At j > 30 A/cm^2^, the p-n junction is open and the injected carriers interact with the entire defect system, including not only EDs and SRH defects but also the extended defect system (dislocations, grain boundaries, V-defects), which increases losses due to nonradiative recombination. At the same time, the transport mechanism shifts from tunnelling to diffusion. These processes are identified using cathodoluminescence and are presented in [46]. The briefly discussed processes do not exclude the involvement of Auger recombination and therefore require further investigation.

Our experimental results on the degradation of UVLEDs during aging have led us to assume that degradation primarily occurs due to the generation of defects in local regions with disrupted alloy stoichiometry in the QWs and at heterointerfaces, rather than solely due to SRH defects, as assumed in most studies, including review articles [4]. This is indicated by the increase in forward and reverse branch currents (Figure 7b) by orders of magnitude and the deterioration in rectifying properties of the p-n junction, as well as the change in the EL spectra of UVLEDs (Figure 7a) during aging, involving the two mechanisms of carrier interaction with EDs that we identified. The involvement of these mechanisms allows us to explain the observed recovery of I-U characteristics and efficiency values on some degraded LEDs after short-term exposure to voltages slightly above the voltage applied during the aging test.

It appears that recombination processes involving EDs may be the cause of the poorly predictable lifespan of nitride-based LEDs. It has been shown that in green and blue LEDs with low efficiency and high DND, the interaction of EDs with injected charge carriers leads to an increase in efficiency during the first 3000 h [47]. However, over time, a wide variety of scenarios is observed, even for LEDs from the same batch, ranging from rapid failure to several thousand hours of operation.

In addition to the poorly predictable lifespan of nitride-based LEDs, as noted in many studies [4], some experimentally observed effects during aging remain unexplained. For instance, these effects are the shorter lifespan of LEDs with high EQE [4,25] and the observed correlation between the aging progression pattern over time and the diffusion process. It seems that these features can be explained in light of our findings that recombination processes vary depending on the current density (particularly when QWs are inside and outside the SCR) and the DND. In this regard, LEDs with high EQE and low DND have significantly lower values of BFP. This means that the processes in QWs located outside the SCR start earlier than in LEDs with higher DND. These processes include a change in the carrier transport mechanism from tunnelling to diffusive, including in the lateral direction, as well as carrier recombination throughout the entire defect system.

An important result of our approach is the estimation of parameters associated with growth regimes (such as the BFP and DND) on one hand, and LED parameters on the other. This contrasts with the consideration of charge carrier recombination processes within the framework of the ABC model, which may hinder understanding and even lead to a loss of important information about the complex physical processes in nitride LEDs.

## 5. Conclusions

Our study revealed a significant correlation between the electrical and optical properties of LEDs emitting in range from 270 to 540 nm with EQE ranging from 4% to 70% and the degree of nanomaterial disorder in QWs and their heterointerfaces. DND depends on the nanoarrangement of domain structure, random alloy fluctuations, and the presence of local regions with disrupted alloy stoichiometry. Our experiment shows that a higher DND correlates with increased ED concentrations and reduced LED EQE.

We identify two mechanisms of interaction between EDs and charge carriers, both of which involve multiphonon carrier capture and ionization processes. The first mechanism provides coordination rearrangement of EDs in the lattice, resulting in narrowed electroluminescence spectra and improved radiative recombination efficiency. The second mechanism, a recombination-induced defect heating process, broadens electroluminescence spectra. Both mechanisms contribute to reversible changes in LED parameters during aging. Additionally, both mechanisms impact EQE and contribute to efficiency droop and EQE reduction with an increase in indium or aluminium content.

The effect of peak EQE reduction (green gap) with increasing indium and aluminium fraction is primarily due to increased DND and corresponding charge carrier recombination features in MQWs located within the SCR of the p-n junction. Additionally, the efficiency droop effect is influenced by recombination in MQWs located outside the SCR after the p-n junction is opened. The efficiency droop level just after p-n junction opening is determined by the magnitude of BFP in QWs beyond the SCR. The decrease in EQE with further increasing current density involves a transition in charge carrier transport mechanisms from tunnelling in the BFP field to diffusive transport. This enhances nonradiative recombination involving the entire defect system, including dislocations and grain boundaries. Experimentally obtained estimates of BFP in QWs, relative to their location with respect to the SCR, correlate with DND and can be used to control growth processes in the buffer layers and active regions of LEDs.

We find that LEDs with lower maximum efficiency exhibit high DND, indicating poorly structured QWs and barriers. This contributes to low EQE values in green and UV LEDs. Consequently, real LEDs encounter more significant sources of charge carrier losses beyond those caused by SRH centers. Disordered heterointerfaces are the most potent source of losses, hindering carrier localization in MQWs. The level of reverse-bias currents serves as a criterion for identifying such LEDs. The DND of nitrides largely determines the characteristics of recombination processes in LEDs. It appears that a more detailed study of the properties of excited defects, alloy disorder, and disrupted heterointerfaces is necessary to address practically important tasks of improving the efficiency of green and UV LEDs.

It can also be one of the reasons for the lack of the long-awaited progress in GaN-based quantum cascade lasers that are expected to overcome their InP, GaAs, and InAs-based counterparts due to deeper QWs and higher optical phonon energy.

It appears that the strong influence of DND in nitride-based light-emitting devices on their parameters necessitates a reconsideration of existing approaches applied to the theoretical analysis of processes occurring during the operation of these devices, as well as their modelling and simulation. Perhaps, in constructing theoretical models of physical processes in nitride-based devices, it is necessary to take into account the DND of nitrides and employ synergistic and fractal methods to characterize the properties of these low-dimensional materials.

## Figures and Tables

**Table 1:** Comparison of parameters of various LEDs.

Title 1	Blue	Green 1	Green 2	Green 3	Green 4
Maximum EQE value (Figure 6a), %	73	38	30	16	12
u_th_-u_0_, (Figure 3b,c), V	0	0.13	0.46	0.67	1
FHWM at 0.3 mA (Figure 6a), nm	15	29.1	31.6	37.5	42
BFP within the SCR (Figure 7b), meV	4	8	12	15	50
BFP outside the SCR (Figure 7b), meV	24	38	50	36	20

## Data Availability

Data is contained within the article.
